# Genome-Wide Analysis of CDPK Family in Foxtail Millet and Determination of *SiCDPK24* Functions in Drought Stress

**DOI:** 10.3389/fpls.2018.00651

**Published:** 2018-07-26

**Authors:** Tai-Fei Yu, Wan-Ying Zhao, Jin-Dong Fu, Yong-Wei Liu, Ming Chen, Yong-Bin Zhou, You-Zhi Ma, Zhao-Shi Xu, Ya-Jun Xi

**Affiliations:** ^1^State Key Laboratory of Crop Stress Biology for Arid Areas, Northwest Agricultural and Forestry University, Yangling, China; ^2^Institute of Crop Science, Chinese Academy of Agricultural Sciences, National Key Facility for Crop Gene Resources and Genetic Improvement, Key Laboratory of Biology and Genetic Improvement of Triticeae Crops, Ministry of Agriculture, Beijing, China; ^3^Institute of Genetics and Physiology, Hebei Academy of Agriculture and Forestry Sciences, Plant Genetic Engineering Center of Hebei Province, Shijiazhuang, China

**Keywords:** calcium-dependent protein kinase (CDPK), expression pattern, gene regulation, drought resistance, foxtail millet

## Abstract

Plant calcium-dependent protein kinases (CDPKs) were reported to play important roles in plant resistance to abiotic stress. Foxtail millet cultivation “H138” was used for RNA-seq analysis. The data from drought-induced *de novo* transcriptomic sequences of foxtail millet showed that CDPKs were up- or down-regulated by drought to different degrees. In this study, 29 foxtail millet CDPKs were classified into four subgroups. These genes were unevenly distributed on nine foxtail millet chromosomes, and chromosomes 2, 3, and 9 contained the most SiCDPK members. Analysis of putative *cis*-acting elements showed that most foxtail millet CDPK genes contained the ABRE, LTR, HSE, MYB, MYC, DRE, CGTCA-motif, and TGACG-motif *cis*-acting elements, which could be activated by abiotic stresses. Real-time PCR analysis indicated that 29 SiCDPK genes experienced different degrees of induction under drought and ABA stresses. *SiCDPK24* had the highest expression levels at 6 and 12 h of drought treatment and was chosen for further analysis. SiCDPK24 localized to the cell membrane and the nucleus of *Arabidopsis* mesophyll protoplasts. Western blot analysis showed that SiCDPK24 protein had autophosphorylation activity. Overexpression of *SiCDPK24* in *Arabidopsis* enhanced drought resistance and improved the survival rate under drought stress. It also activated the expressions of nine stress-related genes, namely *RD29A*, *RD29B*, *RD22*, *KIN1*, *COR15*, *COR47*, *LEA14*, *CBF3/DREB1A*, and *DREB2A*. These genes are involved in resistance to abiotic stresses in *Arabidopsis*. These results indicate that foxtail millet CDPK genes play important roles in resisting drought stress.

## Introduction

Abiotic stresses, such as heat, cold, drought, and salt, often affect plant growth and metabolic processes, and can dramatically reduce crop yield. Plants have developed a variety of sophisticated strategies to resist abiotic stresses (Bohnert et al., 1995; Bray, 1997; Zhu et al., 1997). Many stress-responsive genes which can produce or regulate enzymes involved in the biosynthesis of various osmoprotectants, late embryogenesis abundant and glutathione *S*-transferase proteins are induced in plants to counteract the environmental damage (Saijo et al., 2000). Transferring these genes into plants can be used to verify the important roles of induced stress-responsive genes in resisting abiotic stresses (Holmberg and Bülow, 1998).

Calcium-dependent protein kinases (CDPKs) are important with regard to the abiotic stress resistance of plants. They are affected by the calcium (Ca^2+^) levels in plant cells. Ca^2+^ plays important roles in a variety of cellular and physiological processes as a second messenger (Ghosh and Greenberg, 1995; Rudd and Franklin-Tong, 2001). The Ca^2+^ concentration can be rapidly increased by hormones, light, mechanical disturbances, abiotic stresses, and pathogen elicitors (Sanders et al., 1999; Rudd and Franklin-Tong, 2001). The change of Ca^2+^ concentration in plant signals is mediated and transferred by combining with protein phosphorylation or dephosphorylation cascades (Saijo et al., 2000). The Ca^2+^ influx depends on intracellular protein mediators, such as calmodulin (CaM), CDPKs, and calcineurin B-like proteins. Among these intracellular protein mediators of the Ca^2+^ influx, Ca^2+^-stimulated protein phosphorylation is performed predominantly by the members of the CDPK family in plants (Trewavas and Malho, 1997; Sanders et al., 1999). The CDPK family contains a calmodulin-like regulatory domain with four or fewer EF hands, which are Ca^2+^-binding sites, at the C-terminal end, which can be activated directly through Ca^2+^ binding (Roberts and Harmon, 1992). They also contain a variable N-terminal domain (often with myristoylation or palmitoylation sites associated with subcellular targeting), a conserved serine/threonine kinase domain, and an auto-inhibitory junction region (Cheng et al., 2002).

CDPKs are a very large family in plants. The first *CDPK* gene was found and isolated from soybean (Harper et al., 1991). Since then, genes encoding CDPKs have been identified in a wide variety of plant species. Of these, 34 CDPK genes are found in *Arabidopsis*, 31 in rice, 40 in maize, and 20 in wheat (Cheng et al., 2002; Asano et al., 2005). CDPKs are widely distributed in plant tissues such as the root, stem, leaf, flower, and fruit; they have different functions and participate in multiple different signaling pathways (Romeis et al., 2001). *AtCPK17* and *AtCPK34* are mainly located in mature pollen to regulate the elongation of the pollen tube (Myers et al., 2009). CDPKs also participate in hormone regulation and stress responses. *AtCPK10* is involved in Ca^2+^-mediated regulation of stomatal movements in *Arabidopsis* under drought treatment and plays important roles in ABA signaling (Zou et al., 2010). *OsCDPK13*, a CDPK gene from rice, is induced by cold and gibberellin treatment (Abbasi et al., 2004). CDPKs also play important roles in disease resistance. *StCDPK5* confers resistance to late blight pathogen in transgenic potato plants (Kobayashi et al., 2012). The functional diversity of the CDPK family allows one or more of its members to have a role in every stage of plant growth and development.

Foxtail millet (*Setaria italica*) belongs to the poaceae grass family that is widely cultivated in northern China and India. It is an important food and fodder crop in arid regions and has potential for use as a C4 biofuel. The functions of CDPKs are still unknown in foxtail millet. Foxtail millet cultivation “H138” has been used for RNA-seq analysis and reported to have strong drought resistance (Chen, 2013). We analyzed the foxtail millet whole genome and found 29 CDPK family members. We identified a multiple stress-responsive CDPK gene, and the expression of all 29 CDPK family members was assessed in response to drought. Real-time PCR analysis revealed that *SiCDPK24* had the highest transcript levels after drought treatment and the gene was chosen for further analysis. *SiCDPK24* responded to different stresses and was induced by drought, salt, heat, ABA, and cold. Overexpression of *SiCDPK24* in *Arabidopsis* plants improved tolerance to drought stress.

## Materials and Methods

### RNA-Seq Analysis

Four-leaf-stage untreated foxtail millet seedlings were washed off with water, and then the seedlings were dehydrated on a piece of filter paper for 2 h. The samples were collected for RNA-seq analysis. The total RNA of foxtail millet was extracted using the TRIzol reagent (Invitrogen, Carlsbad, CA, United States) following the manufacturer’s instructions. The NanoPhotometer spectrophotometer (IMPLEN, Westlake Village, CA, United States) and the RNA 6000 Nano Assay Kit for the Agilent 2100 Bioanalyzer system (Agilent Technologies, Santa Clara, CA, United States) were used to assess RNA purity and integrity. After assessment, RNA purity was cleaved by using divalent cations in the NEBNext First Strand Synthesis reaction buffer under elevated temperature, and then first-strand cDNA was synthesized from the cleaved RNA fragments using reverse transcriptase and random primers. Subsequently, the double-stranded cDNA synthesis was carried out exploiting DNA polymerase I and RNase H. The cDNA library was prepared and then sequenced by Allwegene Technology Company in Beijing, China. The Illumina HiSeq 4000^TM^ platform (Illumina, Inc., San Diego, CA, United States) was used for sequencing all libraries. Clean reads were acquired after steps of raw sequence data processing. Before mapping the sequencing reads, adapter sequences were filtered from the raw reads. To obtain more reliable results, low-quality reads (>50% bases with quality scores ≤5) and unknown bases (>10% N bases) were filtered from each dataset. The trinity program was used for the *de novo* transcriptome assembly of the high-quality clean reads (Grabherr et al., 2011).

The sequencing reads were mapped to the foxtail millet reference genome using TopHat (Trapnell et al., 2009). To analyze and optimize the gene structure, the mapped sequencing reads were compared against known gene sequences using Cufflinks (Trapnell et al., 2012). The fragments per kilobase of exon model per million mapped reads (FPKM) algorithm was used for analyzing gene expression levels, as it is a commonly used method to measure the level of gene expression (Mortazavi et al., 2008). The DESeq package (ver. 2.1.0) (Anders and Huber, 2010) was used to detect DEGs between the “Control” and “Drought treatment” samples. The corrected *p*-value (*q*-value) ≤ 0.05 and |log_2_ (fold change)| > 1 were used as the threshold for identifying significant differences in gene expression (Deng et al., 2006). All optimized DEGs were compared with the database of nonredundant protein (NR), the cluster of orthologous groups, the kyoto encyclopedia of genes and genomes (KEGG) pathway, and the gene ontology (GO). Subsequently, the DEG functional classification and enrichment analyses to view the distribution of gene functions were performed by using GOseq and KOBAS software. The methods of RNA-seq were described in the paper by Xiao et al. (2017).

### Identification of CDPKs in Foxtail Millet, *Arabidopsis*, and Rice

According to the CDPK domain amino acid sequences of *Arabidopsis*, all of the candidate CDPK genes were obtained from the foxtail millet database (from JGI Glyma1.0 annotation). The gene sequences and protein sequences of *Arabidopsis* and rice CDPKs were acquired from TAIR (Huala et al., 2001) and the JGI Glyma1.0 annotation, respectively.

### Chromosome Locations and Phylogenetic Analysis of Foxtail Millet CDPKs

The position of each SiCDPK member was obtained from Phytozome. All CDPKs were located by using MapInspect software on nine foxtail millet chromosomes. All CDPKs in *Arabidopsis*, rice, and foxtail millet were imported into MEGA5.0, and multiple sequence alignments were performed using Clustal X 2.0. The alignment file was subjected to create an unrooted phylogenetic tree based on the neighbor-joining method; after the bootstrap analysis for 1,000 replicates, the final tree was generated. The method was described in Saitou and Nei (1987).

### Gene Structure, Protein Structure, and *Cis*-Acting Element Analysis of Foxtail Millet CDPKs

The genome sequence and the protein sequence of all CDPKs of foxtail millet were downloaded from Phytozome. The gene structure was predicted using the online Gene Structure Display Server^1^ (Guo et al., 2007). The protein structure was displayed by ExPAsy-PROSITE^2^. We downloaded approximately 2,000 bp upstream of the initiation codon as the promoter for each CDPK from Phytozome. The *cis*-acting elements in these promoter sequences were predicted by using the online database New PlantCARE^3^.

### qRT-PCR Analysis

Four-leaf-stage foxtail millet seedlings growing under normal conditions were treated with 10% PEG6000 and 100 μM ABA, and then we took samples at 0, 3, 6, and 12 h. The samples were saved at -70°C after freezing in liquid nitrogen. The total RNA was isolated from the whole plants by using TRIzol reagent (TaKaRa, Japan) according to the manufacturer’s handbook. First-strand cDNA was synthesized with a PrimeScript First Strand cDNA Synthesis kit (TaKaRa, Japan). Quantitative real-time PCR (qRT-PCR) of the foxtail millet CDPKs was performed with the SYBR Premix ExTaq^TM^ kit (TaKaRa) and an ABI 7500 according to the manufacturer’s protocols (Applied Biosystems). The amounts of transcripts accumulated for SiCDPK genes normalized to the internal control *Actin* (Si001873) were determined using the 2^-ΔΔCt^ method. The amount of WT transcripts accumulated after 0 h of drought and ABA stresses was taken as 1.0. The foxtail millet CDPK gene primers for qRT-PCR were designed using Primer Premier 5.0 software, avoiding the CDPK conserved domains (Supplementary Table S4). To obtain reproducible results, each experiment was repeated three times.

### Subcellular Localization and Autophosphorylation Analyses of SiCDPK24

The protein of SiCDPK24 without a terminator was fused to the N-terminus of the hGFP gene under the control of the CaMV 35S promoter using the specific primers for SiCDPK24-hGFP (In-Fusion^®^ HD Cloning Kit, TaKaRa). Transient expression of the SiCDPK24-hGFP fusion construct and the hGFP control vector in *Arabidopsis* protoplast cells was performed as in Liu et al. (2013).

*SiCDPK24* was inserted into the prokaryotic expression vector pcold (TaKaRa) by using a pair of specific primers (Supplementary Table S4). TF-His-SiCDPK24 was expressed in *Escherichia coli* (BL21) by using isopropyl-β-d-thiogalactopyranoside (IPTG; 1 mM) and was purified by employing standard procedures using Ni beads (GE Healthcare). In brief, 50 ml of BL21 cells containing TF-His-SiCDPK24 was grown in liquid LB culture to an OD_600_ of 0.5–0.6, and then isopropyl-β-d-thiogalactopyranoside (IPTG; 1 mM) was added to the LB, which was left overnight at 16°C to induce protein expression. The method was previously described by Liu et al. (2013).

TF-His-SiCDPK24 was expressed in BL21 cells and purified using a Ni affinity gel. After elution from the beads, the protein solution was dialyzed against a 1,000× volume of buffer containing 25 μM calcium, 20 mM Tris-HCl, pH 7.5, 10 mM MgCl_2_, 10 mM β-mercaptoethanol and 10 μM [γ-^32^P]-ATP (specific activity 500 cpm pmol^-1^), 20 mM MOPS, and 1 mM dithiothreitol (DTT). Reactions were stopped with the addition of cracking buffer and were then boiled for 3 min. After 10 or 12% polyacrylamide gels (SDS-PAGE), proteins were separated. The polyacrylamide gel with the proteins was electroblotted onto nitrocellulose membranes. The membranes were blocked with 5% bovine serum albumin (BSA) for 1 h. After 3 min of washing with TBST, the membranes were transferred to 10 mL antibody diluent, and phospho-antibody (phospho-antibody Tyr/Ser/Thr) was added to antibody diluent (1:1000). After 10 h of induction, the membranes were washed with TBST for 3 min, addition of 10 mL antibody diluent and second antibody (1:2000, goat anti-rabbit antibody). After 1.5 h of induction, the membranes were washed three times with TBST, and every time washed for 3 min and then exposed to excitation light of 700 nm to observe.

### Screening of Transgenic *SiCDPK24* *Arabidopsis*

*Arabidopsis thaliana* ecotypes Col-0 (wild type) under normal conditions (light 16 h/dark 8 h, 23°C, 50% of relative humidity) grew until flowering. *Agrobacterium tumefaciens* strain (EHA105) carrying recombinant vector *SiCDPK24*-pBI121 (kanamycin) was prepared for transformation, and then transferred into the 50 mL liquid LB culture medium with antibiotics (rifampicin and kanamycin) and grown at 28°C. As the OD reaches 0.7–0.8, the *A. tumefaciens* culture medium was spun down and resuspended in 1/2MS fluid medium freshly made in autoclaved MQ, and then Silwet L-77 was added to a concentration of 0.05% (500 μL/L) before dipping and mixed well. The above-ground parts of plant were dipped in *Agrobacterium* solution for 3 min, with gentle agitation. A film of liquid coating the plants was then visible. Dipped plants were placed under dark conditions for 16–24 h to maintain high humidity. Plants were watered and grown normally until the seeds became mature. Dry seeds were harvested and transfomiants were selected by plating in half-strength MS plates containing kanamycin.

### Effects of Drought and ABA on Seed Germination

Wild-type (WT) and T3 homozygous transgenic seeds were surface sterilized, kept at 4°C for 3 days, and then sown on a germination medium. For the germination assay, seeds were placed on the germination medium with 6% or 10% (w/v) polyethylene glycol (PEG6000) 6000 (to simulate osmotic stress) and 0.5 μM or 1 μM ABA. After 3 days of vernalization, the seeds were transferred to normal conditions for germination. The percentage of germinated seeds was calculated based on the number of seedlings at 12, 24, 36, 48, 60, and 72 h. To obtain reproducible results, each experiment was repeated three times.

### Effect of Drought Stress on Transgenic *Arabidopsis* Growth

For the drought stress assay, T3 homozygous transgenic and WT seeds were placed on MS agar plates for germination, kept at 4°C for 3 days, and then transferred to normal conditions for growing. After 7 days of growth, the seedlings from each line were carefully transferred to new MS agar plates supplemented with 6% or 10% PEG6000. After 7 days of upright growth in the treatment medium, seedling root lengths, root surface area, and fresh weight were measured. Three-week-old T3 homozygous transgenic and WT *Arabidopsis* seedlings grown under a 16 h:8 h light:dark cycle with an average daytime temperature of 22°C was used in phenotype experiments. To impose drought, water was withheld for 2 weeks, followed by full rewatering and recovery. Two weeks later, the survival rate was calculated. To calculate the water loss rate, the leaves of 4-week-old T3 homozygous transgenic and WT *Arabidopsis* seedlings were excised and placed on a bench (30% relative humidity), and the fresh weights of plants were measured at 0, 0.5, 2, 3, and 5 h. The fresh weight measured at 0 h was used as the initial weight (*W*_0_), and the fresh weights measured at 0.5, 2, 3, and 5 h were used as *W_n_* for different *n*. The water loss rate was evaluated as (*W*_0_ – *Wn*)^∗^100/*W*_0_.

### Differential Expression of Stress-Responsive Genes in Transgenic and WT *Arabidopsis* Plants

The differential expression of stress-responsive genes was detected in transgenic and WT *Arabidopsis* plants by RT-PCR. Four-week-old untreated transgenic and WT *Arabidopsis* plant seedlings (growing under normal conditions) were washed off with water and dehydrated on filter paper for 6 h, and then the samples were collected for RNA extraction. The total RNA was isolated from the whole plants by using TRIzol reagent (TaKaRa, Japan) according to the manufacturer’s handbook. First-strand cDNA was synthesized with a PrimeScript First Strand cDNA Synthesis kit (TaKaRa, Japan). qRT-PCR of stress-responsive genes was performed with the SYBR Premix ExTaq kit (TaKaRa) and an ABI 7500 according to the manufacturer’s protocols (Applied Biosystems). The amounts of transcripts accumulated for the stress-responsive genes normalized to the internal control *Actin* (At3g18780) were determined using the 2^-ΔΔCt^ method. The stress-responsive gene primers for qRT-PCR are listed in (Supplementary Table S5). To obtain reproducible results, each experiment was repeated three times.

## Results

### RNA-Seq Analyses of Drought-Induced Foxtail Millet Seedlings

To find stress-responsive genes and study how these functional genes worked under stress treatments, four-leaf-stage foxtail millet seedlings were exposed to drought treatment for 2 h, and then the experiment of *de novo* transcriptomic sequencing was started. The results showed that a large number of genes had differential expression before and after the treatment (Supplementary Figure S1A). The differential expression genes were divided into functional groups using GO analyses. More than 40 functionally enriched GO terms were identified to have differential expression genes, and the top 46 enriched GO terms are shown in Supplementary Figure S1B. The distribution of enriched GO terms focused on biological processes related to the response to the stimulus, signaling, and immune system process. The top 20 enriched pathways for the up-regulated genes were shown, with the “Ribosome” pathway that enriched the most differentially expressed genes (Supplementary Figure S1C). From the data of drought-induced *de novo* transcriptomic sequences, the whole family of 29 foxtail millet CDPK genes was detected and showed different expression patterns (Supplementary Figure S1D and Supplementary Table S1). Therefore, we selected the CDPK gene family for further analysis.

### Identification and Analysis of CDPK Genes in Foxtail Millet

**FIGURE 1:**
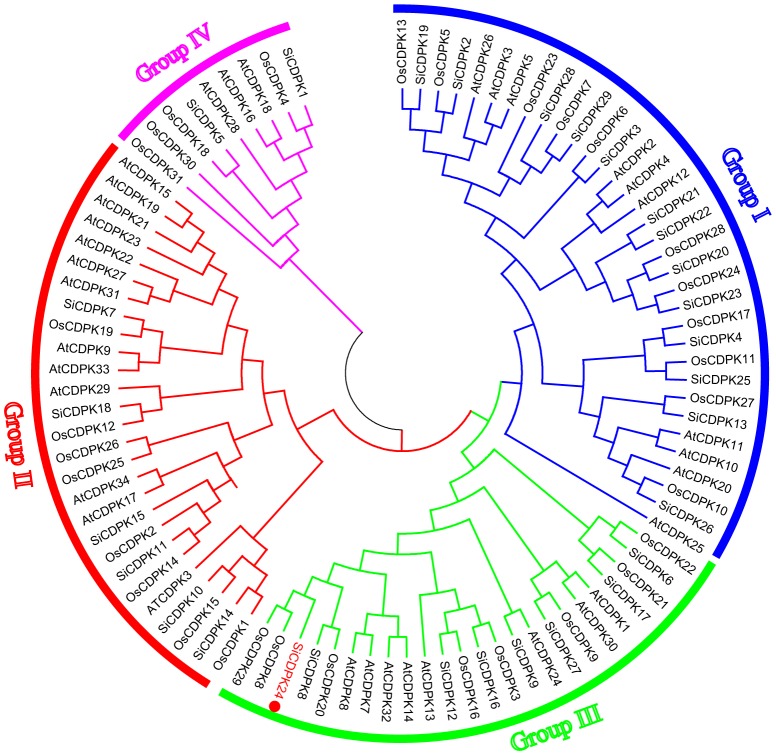
Phylogenetic relationships of CDPKs with *Arabidopsis* *thaliana*, rice, and foxtail millet. The phylogenetic tree is produced by MEGA 5.0 software based on the comparison of amino acid sequences of CDPKs. The neighbor-joining method was used and the bootstrap values were set at 1,000. The frequency values (%) higher than 50 were shown nearby the branch lines. SiCDPKs were divided into four classes, and SiCDPK24 was classified as group III.

**FIGURE 2:**
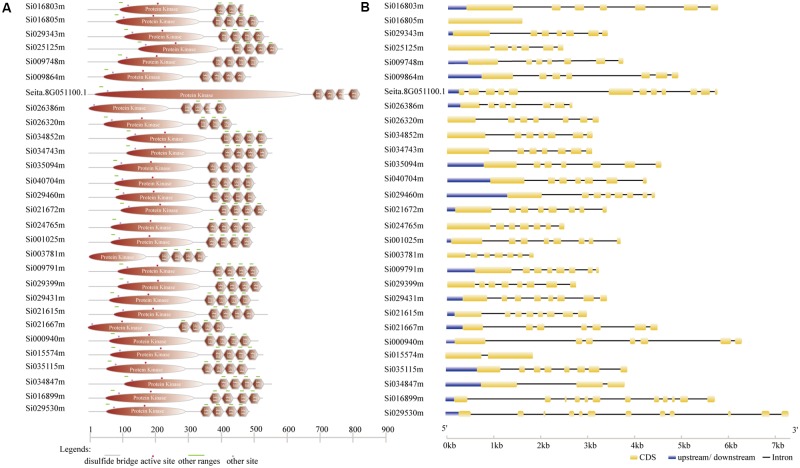
Gene structure analysis of foxtail millet SiCDPKs. **(A)** Protein structure analysis of foxtail millet CDPK genes. **(B)** Intron–exon structures of foxtail millet CDPK genes.

**FIGURE 3:**
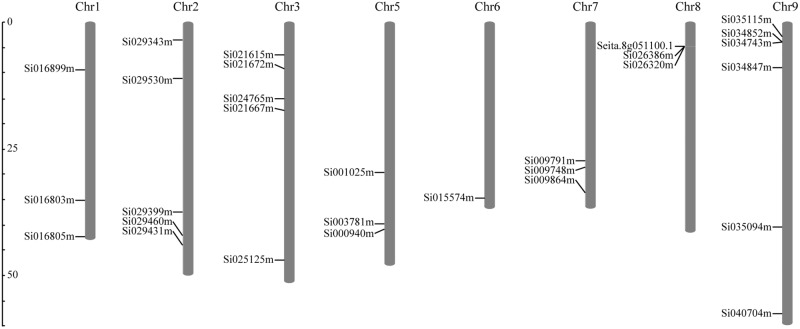
Distribution of SiCDPK genes in foxtail millet genome. The SiCDPK genes distribute on the eight chromosomes.

According to the completed foxtail millet genome sequence, 29 foxtail millet CDPK genes were identified (Supplementary Table S2). To detect the evolutionary relationships between them, a neighbor-joining tree was generated from alignments of the full-length sequences of rice, foxtail millet, and *Arabidopsis* CDPK genes. As shown in **Figure 1**, 29 foxtail millet CDPK genes were clustered into four distinct groups. Most foxtail millet CDPK genes were focused in Group I, which contained 13 foxtail millet CDPKs. Group II contained six foxtail millet CDPKs, Group III had eight, and Group IV had two (**Figure 1**). Gene structure analysis showed that most of the foxtail millet CDPKs had six or seven introns (**Figure 2B**), and the protein structure showed that the 29 foxtail millet CDPKs had conserved domains, such as those encoding protein kinase domains and EF hand domains (**Figure 2A**). The 29 foxtail millet CDPK genes were distributed among nine chromosomes, the only exception being chromosome 4 (**Figure 3**). Among these nine chromosomes, chromosomes 2, 3, and 9 contained the most CDPK genes (5, 5, and 6, respectively) (**Figure 3**).

### *Cis*-Acting Elements Located in Foxtail Millet CDPK Gene Promoters

*Cis*-element analysis showed that every foxtail millet CDPK gene promoter contained MYB and MYC elements, and most foxtail millet CDPK genes had CGTCA-motif and TGACG-motif *cis*-acting elements (Supplementary Table S3). In addition, 66% of the foxtail millet CDPK gene members contained an ABA-responsive element (ABRE), 35% of the members contained a low-temperature response element (LTR), and 45% contained a heat shock response element (HSE) (Supplementary Table S3). Among the 29 foxtail millet CDPK genes, only *SiCDPK1*, *SiCDPK4*, *SiCDPK5*, *SiCDPK7*, *SiCDPK17*, and *SiCDPK21* did not contain a dehydration response element (DRE). These results indicated that most foxtail millet CDPK genes might play an important role in the abiotic stress responses.

### Expression Pattern Analysis of *SiCDPKs* Under Drought and ABA Stresses

**FIGURE 4:**
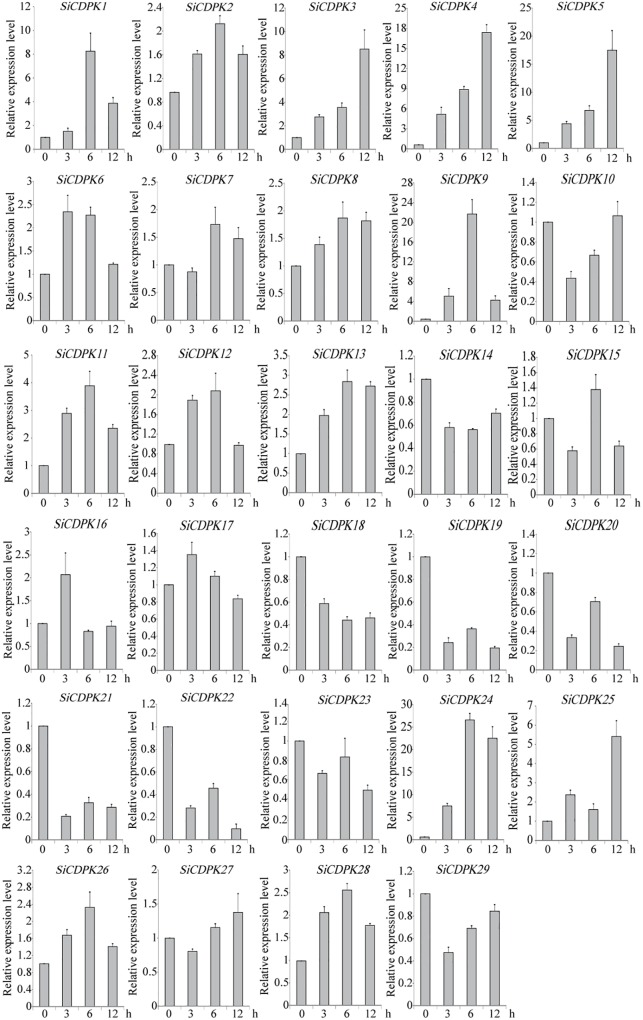
Expression patterns of SiCDPK genes under drought treatment. qRT-PCR data are normalized using foxtail millet *Actin* (Si001873) gene and shown relative to 0 h. The *x*-axes show time periods and the *y*-axes are scales of the relative expression level (error bars indicate SD).

**FIGURE 5:**
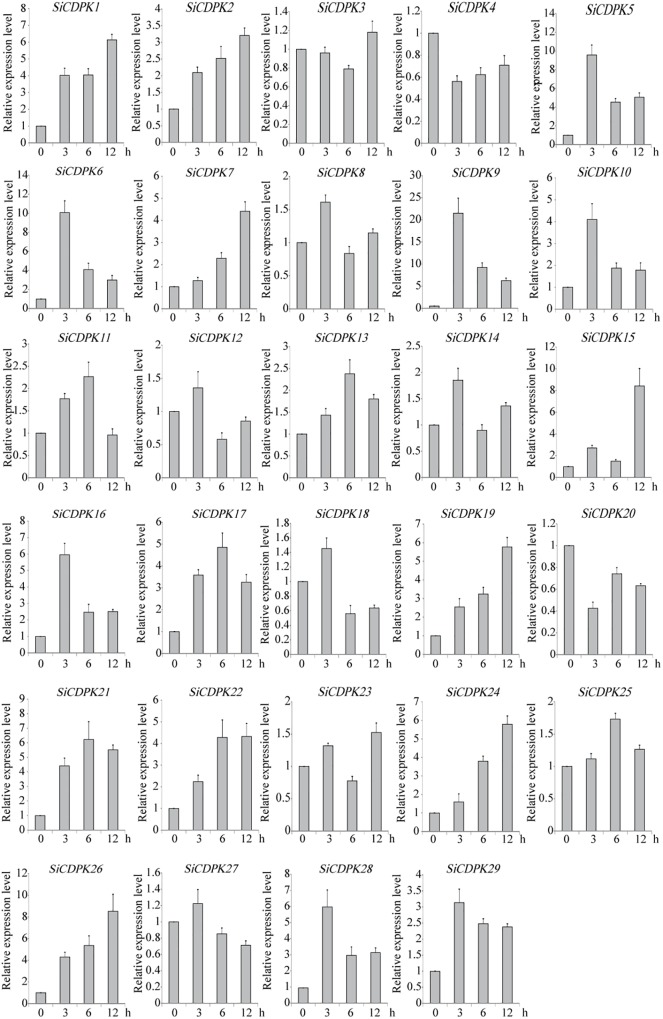
Expression patterns of SiCDPK genes under ABA treatment. qRT-PCR data are normalized using foxtail millet *Actin* (Si001873) gene and shown relative to 0 h. The *x*-axes show time periods and the *y*-axes are scales of the relative expression level (error bars indicate SD).

**FIGURE 6:**
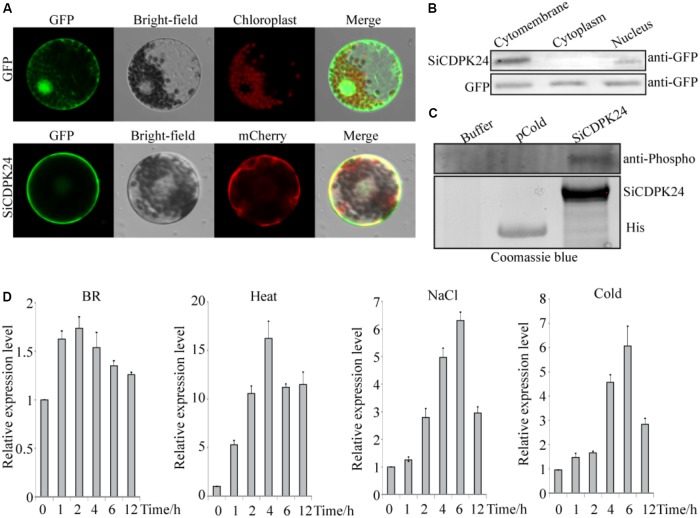
Molecular characteristics of *SiCDPK24* gene. **(A)** Subcellular localization analysis of SiCDPK24 protein. **(B)** Western blot detection analysis of SiCDPK24 protein subcellular localization. **(C)** Autophosphorylation analysis of SiCDPK24 protein. **(D)** Expression patterns of *SiCDPK24* under different stresses. Vertical bars in **(D)** indicate ±SE of three replicates.

To analyze the foxtail millet CDPK gene expression at the transcript level under drought, qRT-PCR was carried out by using RNA isolated from stress-treated foxtail millet. Thirteen *SiCDPK* genes were up-regulated by drought (**Figure 4**). Among them, *SiCDPK1*, *SiCDPK3*, *SiCDPK4*, *SiCDPK5*, *SiCDPK9*, *SiCDPK11*, *SiCDPK24*, and *SiCDPK25* had significant increases in the transcript level after drought treatment, especially *SiCDPK4*, *SiCDPK5*, *SiCDPK9*, and *SiCDPK24*, which had a 10-fold increase. Although the expression levels of *SiCDPK2*, *SiCDPK7*, *SiCDPK8*, *SiCDPK12*, and *SiCDPK17* were induced to increase at 6 and 3 h of drought treatment, respectively, no significant differences were found before and after the drought treatment. Eleven *SiCDPK* genes were down-regulated by drought (**Figure 4**). Among them, *SiCDPK14*, *SiCDPK18*, *SiCDPK19*, *SiCDPK20*, *SiCDPK21*, and *SiCDPK22* had significant decreases in the transcript level after the drought treatment. Most *SiCDPK* genes were up-regulated by ABA (**Figure 5**). However, *SiCDPK4* and *SiCDPK20* were down-regulated by ABA, and *SiCDPK3*, *SiCDPK8*, *SiCDPK12*, *SiCDPK14*, *SiCDPK18*, *SiCDPK23*, *SiCDPK25*, and *SiCDPK27* had no significant changes before and after the ABA treatment. By analyzing the expression pattern of SiCDPKs under drought and ABA stresses, we found that *SiCDPK24* had the highest transcript levels at 6 and 12 h of drought treatment and was induced by ABA (**Figures 5, 6**). Therefore, we chose *SiCDPK24* for further analysis.

### Molecular Characteristics of *SiCDPK24* Gene and Its Product

The subcellular localization of the encoded SiCDPK24 protein was determined and assessed by the transient expression assays in *Arabidopsis* protoplasts using translational fusions to GFP. The control GFP localized to the plasma membrane, nucleus, and cytosol, whereas SiCDPK24-GFP localized to the plasma membrane and the nucleus only (**Figure 6A**). To further verify this result, the plasma membrane, cytosol, and nucleus proteins of *Arabidopsis* protoplasts that contained the GFP signal were extracted and detected by the GFP antibody. The results again showed that SiCDPK24-GFP localized to the plasma membrane and the nucleus (**Figure 6B**).

To test its autophosphorylation activity, the coding region of SiCDPK24 was cloned into the pcold expression vector, and the recombinant fusion protein was successfully induced in *E. coli* as an N-terminal TF-His fusion (**Figure 6C**). The assay to detect autophosphorylation activity was carried out using anti-phosphoamino acid antibodies, and the result revealed that SiCDPK24 had autophosphorylation activity, whereas the control TF-His had no detectable autophosphorylation (**Figure 6C**).

To analyze the *SiCDPK24* expression at the transcript level under stress conditions, qRT-PCR was carried out by using the RNA isolated from stress-treated foxtail millet. *SiCDPK24* was induced not only by drought and ABA but also by NaCl, heat, and cold (**Figure 6D**). In response to NaCl and cold, *SiCDPK24* peaked at 6 h after treatment and then dropped off over time. At 6 h of NaCl stress, the *SiCDPK24* transcript levels were up-regulated to 6.48-fold the unstressed level. During cold treatment, the *SiCDPK24* transcript levels were up-regulated by 6.12-fold at 6 h. Under heat stress, the transcription level of *SiCDPK24* increased 16.95-fold at 2 h and then rapidly decreased to a level similar to that of the control. However, the transcription level of *SiCDPK24* had no significant changes under brassinolide treatment (**Figure 6D**).

### Overexpression of *SiCDPK24* in *Arabidopsis* Affects Germination Rates Under Abiotic Stresses

**FIGURE 7:**
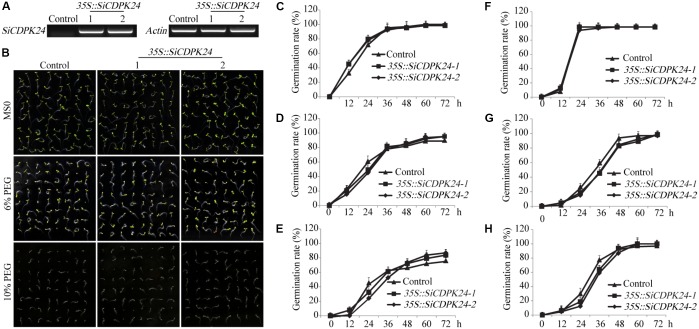
Germination rates of transgenic and WT seeds under PEG6000 and ABA stresses. **(A)** The expression of *SiCDPK24* gene was detected in transgenic and WT plants by using the semi-quantitative PCR method. **(B)** Phenotypes of transgenic and WT *Arabidopsis* plants under different concentrations of PEG6000 treatment. **(C,F)** Germination rates of transgenic and WT *Arabidopsis* seeds on the MS solid medium. **(D,E)** Germination rates of transgenic and WT *Arabidopsis* seeds on the MS solid medium supplemented with different concentrations of PEG6000 treatment (6 and 10%). **(G,H)** Germination rates of transgenic and WT *Arabidopsis* seeds on the MS solid medium supplemented with different concentrations of ABA treatment (0.5 and 1 μM). Vertical bars indicate ±SE of three replicates.

T3 homozygous seeds of the transgenic lines and WT were detected by semi-quantitative PCR (**Figure 7A**) and used for germination. The seeds of WT and the two transgenic *Arabidopsis* lines were placed on half-strength solid Murashige and Skoog (MS) medium supplemented with various concentrations of PEG6000. There was no significant difference in the germination rate between WT and transgenic *Arabidopsis* lines under normal conditions (**Figures 7C,F**). However, under different concentrations of PEG6000 treatment, the germination of WT and transgenic *Arabidopsis* lines was inhibited, and the transgenic *Arabidopsis* lines had lower germination rates than did the WT at 12 and 24 h of germination in 6% and 10% PEG6000 treatment, respectively. After 60 h of germination, although most seeds of WT and the two transgenic *Arabidopsis* lines had germinated, the transgenic *Arabidopsis* lines had higher germination rates than did the WT (**Figures 7B,D,E**). However, under different concentrations of ABA treatment, the transgenic *Arabidopsis* lines had lower germination rates than did the WT at 24, 36, and 72 h of germination (**Figures 7G,H**).

### *SiCDPK24* Improves Drought Stress Tolerances in Transgenic *Arabidopsis*

**FIGURE 8:**
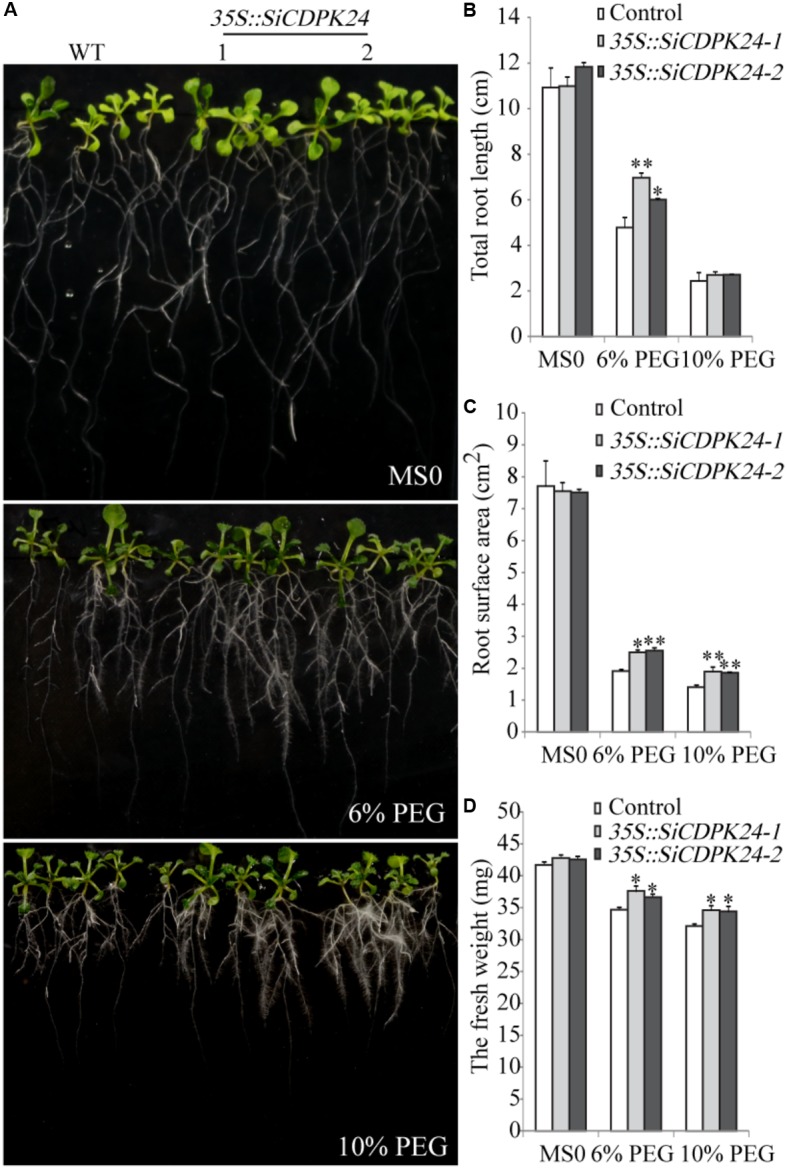
Phenotype identification of transgenic and WT seedlings under PEG6000 stress. **(A)** Growth states of transgenic and WT seedlings under different concentrations of PEG6000 treatment. **(B)** Total root lengths of transgenic and WT *Arabidopsis* seedlings under different concentrations of PEG6000 treatment. **(C)** Root surface areas of transgenic and WT *Arabidopsis* seedlings under different concentrations of PEG6000 treatment. **(D)** Fresh weights of transgenic and WT seedlings under different concentrations of PEG6000 treatment. Vertical bars indicate ±SE of three replicates. ^∗^ and ^∗∗^ indicate significant differences in comparison with the WT lines at 0.01 < *P* < 0.05 and *P* < 0.01, respectively.

To further analyze the functions of *SiCDPK24*, phenotypic analysis was carried out by using homozygous T3 seeds of transgenic lines. Nine-day-old *Arabidopsis* seedlings were transferred to MS plates supplemented with 6% PEG6000 or 10% PEG6000. After a week of PEG6000 treatment, the total root lengths of control plants were 33.7% (6% PEG6000) and 9.8% (10% PEG6000) shorter than those of the *SiCDPK24* transgenic lines. The *SiCDPK24* transgenic lines showed fresh weight reductions of 11% (6% PEG6000) and 17% (10% PEG6000) (average of the two transgenic plant lines), compared to 17% (6% PEG6000) and 22% (10% PEG6000) reductions of the control plants. Under PEG6000 treatment, the root surface area of the *SiCDPK24* transgenic lines was 32.3% (6% PEG6000) and 33.9% (10% PEG6000) larger than that of the WT (**Figure 8**).

**FIGURE 9:**
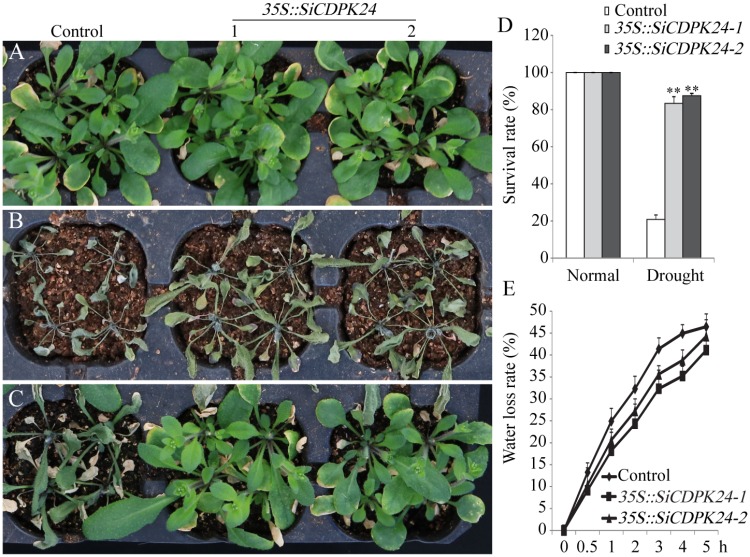
Phenotype identification of transgenic and WT seedlings under different water control conditions. **(A)** Growth states of transgenic and WT seedlings under normal irrigation condition. **(B)** Growth states of transgenic and WT seedlings under water-deficient condition. **(C)** Growth states of transgenic and WT seedlings after 7 days of rewatering. **(D)** Survival rates of transgenic and WT *Arabidopsis* seedlings after 7 days of rewatering. **(E)** Water loss rates of the leaves of transgenic and WT *Arabidopsis* seedlings. Vertical bars in **(C,E)** indicate ±SE of three replicates. ^∗∗^ indicates significant differences in comparison with the WT lines at *P* < 0.01.

To evaluate the tolerance of transgenic *Arabidopsis* plants to drought stress, 3-week-old T3 homozygous transgenic *Arabidopsis* lines and WT plants were examined. Under normal conditions, the two transgenic *Arabidopsis* lines and WT plants grew similarly. However, when the transgenic *Arabidopsis* lines and WT plants were exposed to drought, significant differences appeared. After 15 days of drought treatments, 83.3% of transgenic *Arabidopsis* plants survived, and only 20.8% of WT plants survived (**Figure 9D**). The transgenic *Arabidopsis* plants had a higher survival rate and a lower water loss rate than the WT plants (**Figures 9D,E**).

### *SiCDPK24* Enhanced Expression Levels of Stress-Responsive Genes in *Arabidopsis*

**FIGURE 10:**
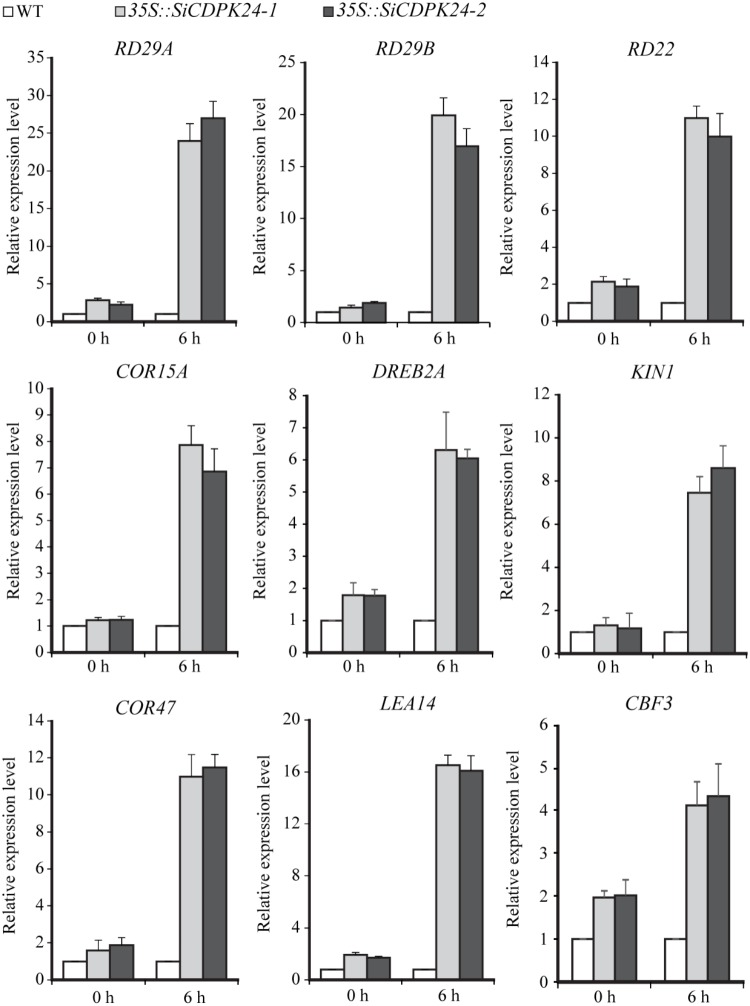
Expressions of stress-responsive genes in transgenic and WT seedlings before or after drought treatment. The transcript levels of stress-related genes in transgenic *Arabidopsis* and WT plants were detected by qRT-PCR. Vertical bars indicate ±SE of three replicates.

To analyze the possible molecular mechanisms of *SiCDPK24* during the stress response, transgenic *Arabidopsis* lines and WT plants were used for analyzing the differential expressions of nine stress-responsive genes, namely, *RD29A*, *RD29B*, *RD22*, *KIN1*, *COR15A*, *COR47, LEA14, CBF3/DREB1A*, and *DREB2A*. These genes act either directly or indirectly in abiotic stress responses. qRT-PCR assays of these six stress response-related genes were performed. A two-fold change in expression was arbitrarily considered expression induction. The qRT-PCR data showed that some stress-responsive genes were up-regulated in the two transgenic *Arabidopsis* lines compared with the control plants under normal growth conditions. However, some stress-responsive genes presented dramatic up-regulation under drought treatment compared with the control plants (**Figure 10**).

**FIGURE 11:**
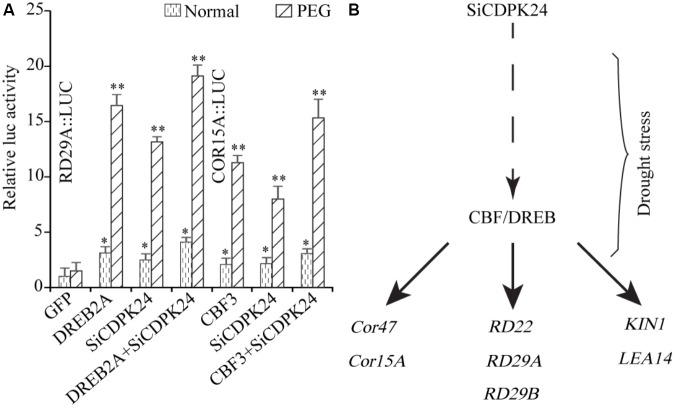
Network prediction model of SiCDPK24 gene regulation. (A) Relative LUC activities analysis. (B) SiCDPK24 gene indirectly increases the expressions of DREB1A and DREB2A, resulting in the activation of the expressions of stress-responsive genes.

To further analyze the regulating function of *SiCDPK24*, the experiment of relative LUC activity detection was implemented. The promoters of *RD29A* and *COR15A* were cloned to analyze the regulating function of *SiCDPK24*. The recombinant vectors were constructed with the promoters of *RD29A* and *COR15A*, which contained the LUC report gene. We transferred the recombinant vectors with CBF/DREB or SiCDPK24 proteins into *Arabidopsis* protoplast cells, respectively. The result showed that the relative LUC activities of the recombinant vectors containing the promoters of *RD29A* and *COR15A* were increased when CBF/DREB or SiCDPK24 proteins existed in *Arabidopsis* protoplast cells under PEG treatment, respectively. However, when CBF/DREB and SiCDPK24 proteins coexisted in *Arabidopsis* protoplast cells that contained the recombinant vectors of *RD29A* and *COR15A* promoters, the relative LUC activities were further increased under PEG treatment (**Figure 11A**).

## Discussion

CDPK genes play important roles in plant growth and development and form a large family in plants (Zhang et al., 2015). They have also been found to be important in the responses to biotic and abiotic stresses. CDPK families in different plants have been analyzed. In this study, we performed BLAST-P searches in NCBI and the published foxtail millet genome (JGI Glyma1.0 annotation), and a total of 29 CDPKs from foxtail millet were identified by using *Arabidopsis* and rice CDPK sequences as query sequences. These were designated *SiCDPK1*-*SiCDPK29* according to the proposed nomenclature for CDPK genes (Cheng et al., 2002). The structural conservation and divergence of CDPK genes, resulting in gene family expansion and functional conservation or differentiation, is of great significance for research. Structural characteristics, such as acylation sites, active sites, and intron–exon structures, show the details of gene family expansion and divergence. All 29 CDPKs had conserved CDPK domains, a variable N-terminal domain, a Ser/Thr kinase domain, an autoinhibitory junction domain, and a calmodulin-like domain (**Figure 2A**). Cluster analysis, which was in accord with the evolutionary relationships, indicated that foxtail millet, rice, and *Arabidopsis* CDPKs were divided into four clusters. Cluster I contained 13 foxtail millet CDPK genes, Cluster II contained 6, Cluster III contained 8, and Cluster IV contained 2 (**Figure 1**). Introns play important roles in evolution, growth, and development, and research on the role of introns has progressed significantly in recent years. Studies in mammals, nematodes, insects, fungi, and plants have revealed that the introns not only regulate gene expression but also participate in gene evolution (Rose, 2008). Gene structure analysis showed that most *SiCDPK* genes had six or seven introns, but *SiCDPK1*, *SiCDPK5*, and *SiCDPK21* had 11 introns. Only one of the genes (*SiCDPK3*) did not contain introns, while the gene (*SiCDPK17*) had one intron and another gene (*SiCDPK27*) had two introns (**Figure 2B**). We speculate that these low intron numbers were because many introns have been lost during the evolution of foxtail millet.

The analysis of *cis*-acting elements in the promoter regions of CDPK genes revealed elements related to stress responses, such as ABRE, DRE, LTRE, MYB, MYC, and HSE(Supplementary Table S3). MYB participates in drought, low temperature, salt, ABA, and GA stress responses (Zhu et al., 2005), and MYC participates in drought, salt, and ABA stress responses. ABRE responds to drought and ABA via the combination with ABRE-binding proteins (AREBs) (Li et al., 2012). DRE combines DRE-binding proteins (DREBs), which participate in drought, salt, cold, and ABA (Zhang et al., 2009). LTRE contributes primarily to the low-temperature response and regulation (Maestrini et al., 2009). HSE binds heat shock response elements, leading to the defense against heat stress and even recovery from the damage (Yamamoto et al., 2005). The CGTCA-motif and the TGACG-motif are involved in MeJA responsiveness, which plays important roles in multiple physiological processes, including development, senescence and ripening, secondary metabolism, and the response to diverse environmental stresses (Creelman and Mullet, 1997; Pieterse et al., 1998; Turner et al., 2002; Browse and Howe, 2008; Wang et al., 2011). Most SiCDPK genes had ABRE, LTR, HSE, MYB, MYC, DRE, CGTCA-motif, and TGACG-motif *cis*-acting elements (Supplementary Table S3). Thirteen SiCDPK members contained HSE *cis*-acting elements, and 10 SiCDPK members contained LTRE *cis*-acting elements (Supplementary Table S3). These results suggest that SiCDPK genes play significant roles in the regulation of responses to many abiotic stresses. Foxtail millet transcriptome sequencing showed that the levels of 29 SiCDPK genes in stress treatment and before treatment were different (**Figure 1**). qRT-PCR showed that the expression level of 29 SiCDPK genes were not effected by sampling under normal H_2_O treatment (Supplementary Figure S2) and showed that most SiCDPK genes were induced by drought and ABA (**Figures 5, 6**). However, we also found differences in the transcription patterns of the 29 SiCDPK genes. *SiCDPK1* and *SiCDPK5* from Cluster IV had similar expression patterns: they were both up-regulated by drought and ABA. *SiCDPK9* and *SiCDPK24* from Cluster III were also similarly regulated (**Figures 5, 6**). *SiCDPK20*, *SiCDPK21*, *SiCDPK22*, and *SiCDPK23* belonged to the same subgroup of Cluster I (on the same branch), indicating that they had high homology, and they were all down-regulated by drought. These results indicate that the foxtail millet CDPK genes’ functions have been conserved in some, but not all, ways.

Because it had the highest expression at 6 and 12 h of drought treatment (**Figure 4**), *SiCDPK24* was selected for analysis of its expression patterns under drought and ABA stresses. Molecular characterization analyses showed that SiCDPK24 localized to the nucleus and cell membrane (**Figure 6A**). CDPKs are serine–threonine protein kinases (Sheen, 1996). Their functions rely on phosphorylation, which plays important roles in plant calcium signal transduction and response to osmotic stresses (Chehab et al., 2004). A new study found that potato StCDPK7 protein with autophosphorylation activity could locate in the nucleus and interact with PAL protein, suggesting that the autophosphorylation activity of StCDPK7 protein played important roles in protein location and protein–protein interactions (Fantino et al., 2017). In our study, SiCDPK24 protein was proven to have a role in autophosphorylation (**Figure 6C**), and may be important for its function. *OsCDPK29* responds to multiple stresses and can be induced by drought, cold, and salt stresses (Wan et al., 2007). Sequence analyses showed that *SiCDPK24* had high homology with *OsCDPK29* (**Figure 6D**). These results indicate that these genes are highly conserved in function as well as sequence. Further, the overexpression of *SiCDPK24* in *Arabidopsis* plants enhanced resistance to drought (**Figures 9, 10**). Many transcription factors and other stress-related genes had been identified, providing evidence that plants have developed flexible molecular and cellular mechanisms to tolerate various abiotic stresses. In *Arabidopsis*, the *DREB2A* gene regulates the expression of other stress response genes, such as *RD29A*, *RD29B*, *RD22*, and *LEA14*. The study found that although the expression levels of *COR15*, *COR47*, and *KIN1* were up-regulated in transgenic *DREB2A Arabidopsis*, they were not valid target genes of *DREB2A*. Further analysis found that *AtDREB1A* plays a key role in regulating the expression levels of *COR15*, *COR47*, and *KIN1* (Sakuma et al., 2006). These are all *CBF/DREB* target genes that are up-regulated in response to drought (Sakuma et al., 2006). A recent paper reported that the overexpression of *VaCPK29* in *Arabidopsis* could enhance the expressions of *AtDREB1A* and *AtDREB2A* and improve the tolerance to heat stress, suggesting that CDPK protein kinase could affect the expressions of *CBF/DREB* genes (Dubrovina et al., 2017). In this study, we found that the transcription levels of *AtDREB2A* and *CBF3/DREB1A* were up-regulated after 6 h of drought treatment in the two transgenic *Arabidopsis* lines. Then, the target genes of *CBF/DREB* were detected by qRT-PCR, and the transcription levels of these target genes were dramatically increased in the two transgenic *Arabidopsis* lines, especially after 6 h of drought treatment (**Figure 10**). By analyzing the relative LUC activities of stress-responsive gene promoters, we found that SiCDPK24 protein could enhance the activities of DREB1A and DREB2A (**Figure 11A**). These results indicate that a signal pathway might exist in the DREB/CBF gene regulatory network that is stimulated by *SiCDPK24* (**Figure 11B**). We conclude that the millet CDPK family plays important roles in resisting drought stress, based on our analysis of the characteristics of millet CDPK family members and the functions of the *SiCDPK24* gene.

## Author Contributions

Z-SX coordinated the project, conceived and designed the experiments, and edited the manuscript. T-FY, W-YZ, and J-DF performed the experiments, wrote the first draft, and conducted the bioinformatic work. Y-WL revised and edited the manuscript. MC provided the analytical tools and managed reagents. Y-BZ and Y-ZM coordinated the project. Y-JX contributed with valuable discussions. All authors read and approved the final manuscript.

## Conflict of Interest Statement

The authors declare that the research was conducted in the absence of any commercial or financial relationships that could be construed as a potential conflict of interest.

## Footnotes

1 http://gsds.cbi.pku.edu.cn/

2 http://prosite.expasy.org/

3 http://bioinformatics.psb.ugent.be/webtools/plantcare/html/
