# Supplementary material for: Genome-Wide Analysis of CDPK Family in Foxtail Millet and Determination of SiCDPK24 Functions in Drought Stress
Source: Front Plant Sci. 2018 Jul 26;9:651. doi: 10.3389/fpls.2018.00651 (PMC6071576; doi:10.3389/fpls.2018.00651)
Supplement: Supplementary file 1 [file Presentation_1.pdf]

Table S1 RNA-seq data analyses of 29 foxtail millet CDPK genes

Gene ID	Col-Expr -ession	D-treatment -Expression	Col-RPKM	D-treatment -RPKM	log2 Ratio (D-treatment/Col)	Up-Down-Regulation (D-treatment/Col)	P-value
Si016899m	6	29	0.64662	3.0055	2.21661	Up	0.00011
Si016803m	2	8	0.02535	0.0975	1.94359	Up	0.03354
Si016805m	14	46	1.0484	3.31263	1.65979	Up	5.20E-05
Si029343m	17	143	1.83649	14.8557	3.016	Up	3.48E-25
Si029530m	11	41	0.56638	2.0301	1.84171	Up	4.13E-05
Si029399m	104	88	2.45233	1.99548	-0.2974	Down	0.01545
Si029460m	1465	4519	28.2707	83.8609	1.56869	Up	0.01356
Si029431m	63	190	4.49419	13.0342	1.53616	Up	4.35E-15
Si021615m	69	295	2.20507	9.06594	2.03963	Up	1.05E-32
Si021672m	2	0	0.0568	0.001	-5.8278	Down	0.02356
Si024765m	146	341	4.03474	9.06223	1.16739	Up	1.53E-17
Si021667m	3	14	0.1244	0.55826	2.16598	Up	0.00957
Si025125m	77	176	1.87036	4.11118	1.13623	Up	2.16E-09
Si001025m	1345	1275	28.2356	25.7397	-0.1335	Down	0.01786
Si003781m	2692	2071	85.0069	62.8893	-0.4348	Down	3.86E-25
Si000940m	466	1129	29.7633	69.3438	1.22023	Up	3.47E-58
Si015574m	80	213	2.56204	6.55984	1.35637	Up	3.78E-14
Si009791m	69	14	2.65623	0.51828	-2.3576	Down	1.33E-10
Si009748m	6	0	0.19506	0.001	-7.6078	Down	0.01361
Si009864m	62	47	1.56581	1.14147	-0.456	Down	0.01017
Seita.8G051100.1	229	33	6.59484	0.9139	-2.8512	Down	3.23E-39
Si026386m	153	28	7.1152	1.25219	-2.5064	Down	2.28E-23
Si026320m	204	176	16.7793	13.9211	-0.2694	Down	0.06929
Si035115m	1	9	0.12976	1.12304	3.11351	Up	0.01399
Si034852m	6	39	0.164	1.02515	2.64403	Up	6.02E-07
Si034743m	99	513	2.00288	9.98059	2.31705	Up	4.90E-65
Si034847m	1375	987	21.9242	15.1341	-0.5347	Down	3.61E-19
Si035094m	9	42	0.99365	4.45922	2.16598	Up	3.96E-06
Si040704m	298	107	4.60838	1.59123	-1.5341	Down	8.79E-24

Table S2 The characteristics of millet CDPK family members

Genename	GeneID	Gene locus	N-Myr	N-Pal	No.of EF_S	**pI**	No. of aa	M.W (KDa)
SiCDPK1	Si016899m	Scaffold 1:8840816..8847147	Y	Y	4	8.89	552	58.9
SiCDPK2	Si016803m	scaffold1:4830834..34835537	Y	Y	4	5.59	484	61.4
SiCDPK3	Si016805m	scaffold1:42005655..42007553	N	Y	4	5.37	558	61.1
SiCDPK4	Si029343m	scaffold2:3226078..3230187	N	Y	4	5.54	573	63.1
SiCDPK5	Si029530m	scaffold2:10839583..10847218	N	Y	4	7.65	512	57.6
SiCDPK6	Si029399m	scaffold2:37157233..37160378	Y	Y	4	7.86	553	62.0
SiCDPK7	Si029460m	scaffold2:41740491..41745028	Y	Y	4	5.94	531	59.5
SiCDPK8	Si029431m	scaffold2:43623996..43627879	N	Y	4	6.38	541	60.9
SiCDPK9	Si021615m	scaffold3:6126161..6129363	N	Y	4	5.99	568	63.1
SiCDPK10	Si021672m	scaffold3:8775983..8779648	Y	Y	4	6.53	546	61.5
SiCDPK11	Si024765m	scaffold3:14854877..14857330	N	Y	4	5.6	529	58.2
SiCDPK12	Si021667m	scaffold3:17043761..17048442	NN	Y	4	6.07	457	51.8
SiCDPK13	Si025125m	scaffold3:46584235..46586884	N	Y	4	5.7	618	68.3
SiCDPK14	Si001025m	scaffold5:29354804..29359304	Y	Y	4	6.04	522	59.0
SiCDPK15	Si003781m	scaffold5:39548186..39550182	NN	Y	4	5.25	377	42.5
SiCDPK16	Si000940m	scaffold5:40607910..40614617	Y	Y	4	6.0	541	60.5
SiCDPK17	Si015574m	scaffold6:34338578..34340381	Y	Y	4	6.82	556	60.9
SiCDPK18	Si009791m	scaffold7:27056236..27059947	Y	Y	4	6.09	534	60.1
SiCDPK19	Si009748m	scaffold7:28338539..28343706	Y	Y	4	5.6	556	61.2

SiCDPK20	Si009864m	scaffold7:33248290..33253115	NN	Y	4	5.41	515	56.8
SiCDPK21	Seita.8G051100.1	scaffold8:4338220..4345155	NN	Y	4	5.91	843	94.9
SiCDPK22	Si026386m	scaffold8: 4338508-4341101	NN	Y	4	5.15	435	48.8
SiCDPK23	Si026320m	scaffold8:4404915..4408135	NN	Y	3	5.87	473	52.6
SiCDPK24	Si035115m	scaffold9:2438664..2442949	N	Y	4	6.89	531	59.8
SiCDPK25	Si034852m	scaffold9:3605681..3609558	N	Y	4	5.39	582	63.9
SiCDPK26	Si034743m	scaffold9:3622757..3626164	N	Y	4	5.9	614	67.1
SiCDPK27	Si034847m	scaffold9:8599081..8603512	N	Y	4	9.01	583	64.5
SiCDPK28	Si035094m	scaffold9:40133380..40138172	N	Y	2	4.97	535	60.5
SiCDPK29	Si040704m	scaffold9:57206117..57210503	N	Y	4	5.27	527	59.6

Table S3 The cis-acting element analysis of millet CDPK family member promoters

Gene name	Motifs related to stress response	Motifs related to growth and development
Si016803m	Box-W1, TCA-element, GC-motif, MBS, MYB, MYC, DRE	Skn-1_motif,
Si016805m	ABRE, ACE, ARE, GARE-motif, LTR, TATC-box, TC-rich repeats, TCA-element, TGA-element, MYB, MYC, DRE	GCN4_motif,CAT-box,MBS,Skn-1_motif,as-2-box
Si029343m	ABRE, ACE, Box-W1, CGTCA-motif, ERE, GC-motif, HSE, MBS, TC-rich repeats, TGACG-motif, MYB, MYC	MBSI,Skn-1_motif,as-2-box ,circadian
Si025125m	ACE, GARE-motif, GC-motif, HSE, LTR, TC-rich repeat, TCA-element, MYB, MYC, DRE	CAT-box, GCN4_motif, MBS, O2-site, Skn-1_motif.
Si009748m	GC-motif, LTR, TC-rich repeats, TGA-element, MYB, MYC, DRE	CCGTCC-box,as-2-box
Si009864m	ABRE, C-repeat/DRE, CGTCA-motif, GARE-motif, GC-motif, HSE, TC-rich repeats, TCA-element, TGACG-motif, MYB, MYC, DRE	CCGTCC-box, circadian
Seita.8G051100.1	ABRE, ACE, ARE, Box-W1, CGTCA-motif, CGTCA-motif, LTR, MBS, TC-rich repeats, TCA-element , TGACG-motif, MYB, MYC	O2-site,Skn-1_motif,circadian
Si026386m	ACE, ARE, CGTCA-motif, LTR, MBS, TC-rich repeats, TCA-element, TGA-element, TGACG-motif, MYB, MYC, DRE	GCN4_motif,CCGTCC-box,HD-Zip1,HD-Zip2, O2-site,Skn-1_motif,circadian
Si026320m	ACE, Box-W1, CGTCA-motif, ERE, GARE-motif, GC-motif, LTR, MBS, P-box, TGACG-motif, MYB, MYC, DRE	AuxRR-core,Skn-1_motif,circadian
Si034852m	ABRE, ARE, Box-W1, CGTCA-motif, HSE, MBS, TGA-element, TGACG-motif, MYB, MYC, DRE	CAT-box,CCGTCC-box,O2-site,Skn-1_motif
Si034743m	ABRE, ARE, Box-W1, CGTCA-motif, GC-motif, HSE, MBS, P-box, TCA-element, TGACG-motif, motif IIb, MYB, MYC, DRE	GCN4_motif,HD-Zip 1,HD-Zip 2
Si035094m	ABRE, Box-W1, ERE, GARE-motif, GC-motif, HSE, MBS, TCA-element, MYB, MYC, DRE	GCN4_motif,Skn-1_motif
Si040704m	ABRE, ARE, GARE-motif, GC-motif, HSE, LTR, MBS, motif IIb TC-rich repeats, TCA-element, MYB, MYC, DRE	CCGTCC-box,GCN4_motif
Si029460m	ARE, Box-W1, CGTCA-motif, ERE, GARE-motif, HSE, MBS, TC-rich repeats, TCA-element, TGA-element, TGACG-motif, MYB, MYC	O2-site ,Skn-1_motif,circadian
Si021672m	ABRE, ACE, ARE, Box-W1, CGTCA-motif, GARE-motif, motif IIb, GC-motif, P-box, TCA-element, TGACG-motif, MYB, MYC, DRE	CAT-box,CCGTCC-box,GCN4_motif,O2-site, Skn-1_motif,
Si024765m	ABRE, ACE, CGTCA-motif, GARE-motif, GC-motif, MBS, TGACG-motif, TC-rich repeats, MYB, MYC, DRE	CAT-box ,CCGTCC-box,Skn-1_motif,as1,
Si001025m	ABRE, ARE, CGTCA-motif, GARE-motif, GC-motif, HSE, motif IIb, TCA-element, TGACG-motif, MYB, MYC, DRE	CCGTCC-box,O2-site,circadian,dOCT,
Si003781m	ABRE, ACE, ARE, Box-W1, CGTCA-motif, GARE-motif, motif IIb, GC-motif,, MBS, TGACG-motif, MYB, MYC, DRE	CCGTCC-box,O2-site,OCT,RY-element,Skn-1_motif, circadian,
Si009791m	ABRE, ARE, CGTCA-motif, GARE-motif, GC-motif, HSE, MBS, P-box, TCA-element, TGACG-motif, motif IIb, MYB, MYC, DRE	CAT-box,CCGTCC-box,O2-site,Skn-1_motif
Si029399m	ARE, CGTCA-motif, GARE-motif, GARE-motif, MBS, P-box, TCA-element, TGACG-motif, MYB, MYC, DRE	O2-site,circadian
Si029431m	ABRE, ARE,CGTCA-motif, GARE-motif, GC-motif, HSE, LTR, P-box, TCA-element, TGACG-motif, MYB, MYC, DRE	CAT-box,CCGTCC-box,O2-site,circadian
Si021615m	ABRE, Box-W1, GARE-motif, GC-motif, HSE, MBS, MYB, MYC, DRE	CCGTCC-box,Skn-1_motif
Si021667m	ACE, ARE, CGTCA-motif, ERE, GC-motif, MBS, P-box, motif IIb, TC-rich repeats, TCA-element, MYB, MYC, DRE	CAT-box, CCGTCC-box, MBSI

Si000940m	ABRE, CGTCA-motif, GARE-motif, GC-motif, HSE, P-box, TGACG-motif, MYB, MYC, DRE	CCGTCC-box,GCN4_motif,O2-site,circadian
Si015574m	ABRE, CGTCA-motif, GC-motif, MBS, TGACG-motif, motif IIb, MYB, MYC	CAT-box,CCGTCC-box,Skn-1_motif,circadian
Si035115m	ABRE, ACE, ARE, CGTCA-motif, GARE-motif, GARE-motif, LTR, MBS, TC-rich repeats, TCA-element, TGACG-motif, HSE, DRE	GCN4_motif,O2-site,Skn-1_motif
Si034847m	CGTCA-motif, GC-motif, MBS, TGACG-motif, MYB, MYC, DRE	CCGTCC-box,GCN4_motif,Skn-1_motif
Si016899m	LTR , TCA-element , Box-W1, TCA-element, MYB, MYC	Skn-1_motif,GC-motif,O2-site,as-2-box
Si029530m	ABRE, ARE, Box-W1, CGTCA-motif, GC-motif, MBS, TC-rich repeats, TGACG-motif, MYB, MYC	Skn-1_motif,as-2-box

Table S4 The RT-PCR primers of millet CDPK family members

Gene	Forward primer	Reverse primer
SiCDPK24-zero	GAGTTCTTCCCTTTCAGTCAGTC	TAACCTGATGACCAAAGAGTAAAAG
SiCDPK24-1302	GGGACTCTTGACCATGATGGGCAACTGCTGC	TCAGATCTA CCCATGGCCGTGTCTCAGCGCCGAT
SiCDPK24-GFP	TATCTCTAGAGGATCCATGGGCAACTGCTGC	TGCTCACCATGGATCCCCGTGTCTCAGCGCCGAT
SiCDPK24-Pcold	TACCCTCGAGGGATCCATGGGCAACTGCTGC	GCTTGAATTCGGATCCCCGTGTCTCAGCGCCGAT
SiCDPK1-RT	GAAAGATGCTGCGGTGGTAGT	GAGCAAAATGTAGGTTA
SiCDPK2-RT	CCTGATCGGCCTCTTGATCC	TGTTGTCTATATCCGCCGCC
SiCDPK3-RT	CTTCACGCCCACCTACACAT	GAGCTCCGTGCACAGGTAG
SiCDPK4-RT	CTTCAACTCCGTCTCCCTC	ACTGATAGGTGGTGCCGAACT
SiCDPK5-RT	GTCCGACAGATGCTCAAGGT	ATTCTGGCCCTGACTTACGC
SiCDPK6-RT	AGCTCTTCGACCGCATCTTC	AGTCCTCCGACTTGTTCACG
SiCDPK7-RT	AGGCAAAGCCAGCACATACA	TGCGGATGTCCTCCCTATCT
SiCDPK8-RT	TTTGACCGGAACGAGAGTGG	CAGTCCGTTCCAGCCTTCAT
SiCDPK9-RT	TGGACACCAACAAGGATGGG	GCAGGTGATCCTCACTACGG
SiCDPK10-RT	CACTCGGTCAACCTCGTCAT	ACCGGGCTTGAAGAATACGG
SiCDPK11-RT	ATCTGGAGCGTCGGCGT	GAGGACGTCGTGTGCAGAAA
SiCDPK12-RT	TTTGGCTGTCTCGCTTCACT	TGACCACGTCCATGCTATCG
SiCDPK13-RT	AGACCGAGAACCTCAAGGACAA	TCCATCACCACGTGTACCG
SiCDPK14-RT	GCCAGTTCGGAGTCACCTAC	ATGACGAGGTTGACCGAGTG
SiCDPK15-RT	GGAGGTGCTCAAGAGGAACTAT	CAAGGATGATTGAGGACGTCG
SiCDPK16-RT	CACCTCGTCATGGAGCTCTG	CGGCGAGTTCTCCTTCTTGT
SiCDPK17-RT	GACAAGGACCACAACGGGAA	TGTCGAAGTACCTGAACGCC
SiCDPK18-RT	GAGACCTCAAGCCCGAGAAC	TAATTCCGGCGCAGTACCTC
SiCDPK19-RT	TCTCGTGGCAGCCTTTTCAT	CGCATTGTTCTTCGACCGAC
SiCDPK20-RT	CACTACCGCATCGGGAAGAA	CTCCATGACGAGATGCACGA
SiCDPK21-RT	ACGCAACAGGGAGTGATGAG	CGTAATGGAGCTTGTTGCCG
SiCDPK22-RT	ATGGTTCTGTGGCACCTGAT	CAGCCGCATCCATTAACGTC
SiCDPK23-RT	TCGTTGCCAAGGGTCACTAC	GCGACATAGTAGGGGCTTCC
SiCDPK24-RT	TGATTTTGGGCTGTCCGTGT	ATTGCCTGTGCTACTCCCTG
SiCDPK25-RT	TATGGCCGCGAAGTTGATGT	GAGGCTTGTCAGGAGCAACT
SiCDPK26-RT	TGGCTCCTGATAAGCCGTTG	ATCAGGGCGTAGATCTCGGA
SiCDPK27-RT	AAGCCCGAGAACTTCCTGTA	GCATCGCCTTCTTCTTCAGC
SiCDPK28-RT	AAATGCTCTGCCCTTGTCCA	CGGTGTCGATTGCCTTGAAC
SiCDPK29-RT	TGAAAGGAAGACAGCCGACC	CCTGCCCGTCCTCATAAACA
Actin (Si001873)	GGCAAACAGGGAGAAGATGA	GAGGTTGTCGGTAAGGTCACG

Table S5 The RT-PCR primers of stress responsive genes

Gene	Forward primer	Reverse primer
AtRD29B	AGACAGAGGAGAGAGCAGAG	CGTTGACCACCGAGATAG
AtCOR15A	GCGATGTCTTTCTCAGGAGC	CGAACTGAGTTTTCTGGCCG
AtCOR47	AGGATTCACCAGCTGTCACG	CCTCTTCAGTGGTCTTGGCA
AtRD29A	TATTCGCCGGAATCTGACGG	GATGCCTCACCGTATCCAGG
AtKIN1	CTGGAGCTGGAGCACAACAG	TTGTTCAGGCCGGTCTTGTC
AtLEA14	CTCACCGCGATCCCTAAACC	GGGTCCGGTATCTTCCCCTT
AtRD22	AGGAGCAAACCCTTTCGTGT	CGCAGTTTTGCCTCCGTAAC
AtDREB2A	GGTTGGCCCAATGATGTGGA	CCATCCTTTCCCTCGAGCTG
AtDREB1A	AGAACCAAACAAGAAAACAAGGA	AAGCCGAGTCAGCGAAATT
Actin (At3g18780)	GGCAAGTCATCACGATTGG	CAGCTTCCATTCCCACAAAC

Figure S1

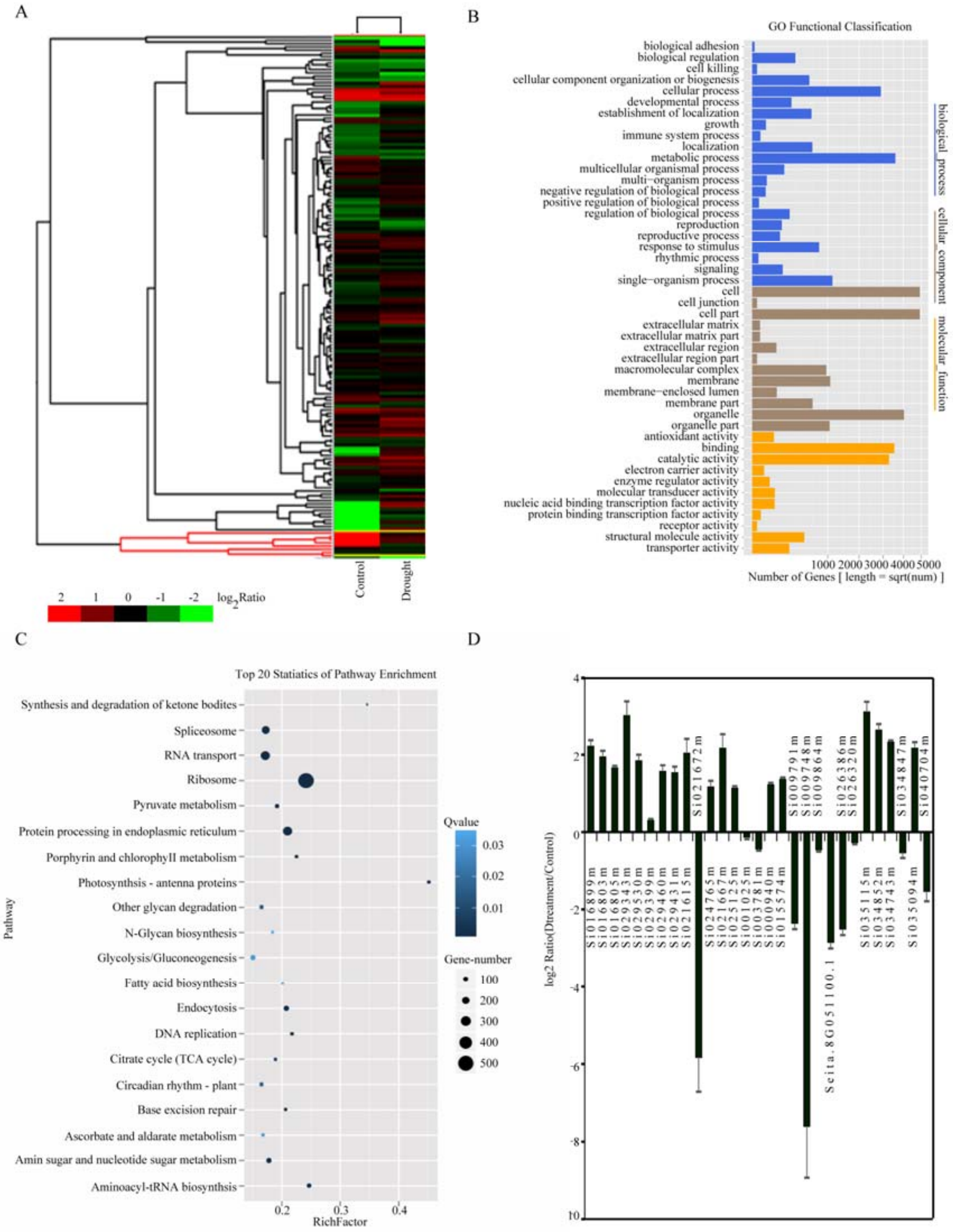

Figure S1 ***De novo*** **transcriptome sequencing analysis**. (**A**) The differential expression of genes before or after drought treatment. **(B)** The most enriched GO terms of up-regulated genes. **(C)** The pathway enrichment statistics of up-regulated genes. (**D**) The expression patterns of all SiCDPK genes after drought treatment in transcriptome sequencing analysis. Vertical bars in (**D**) indicate ±SE of three replicates

Figure S2

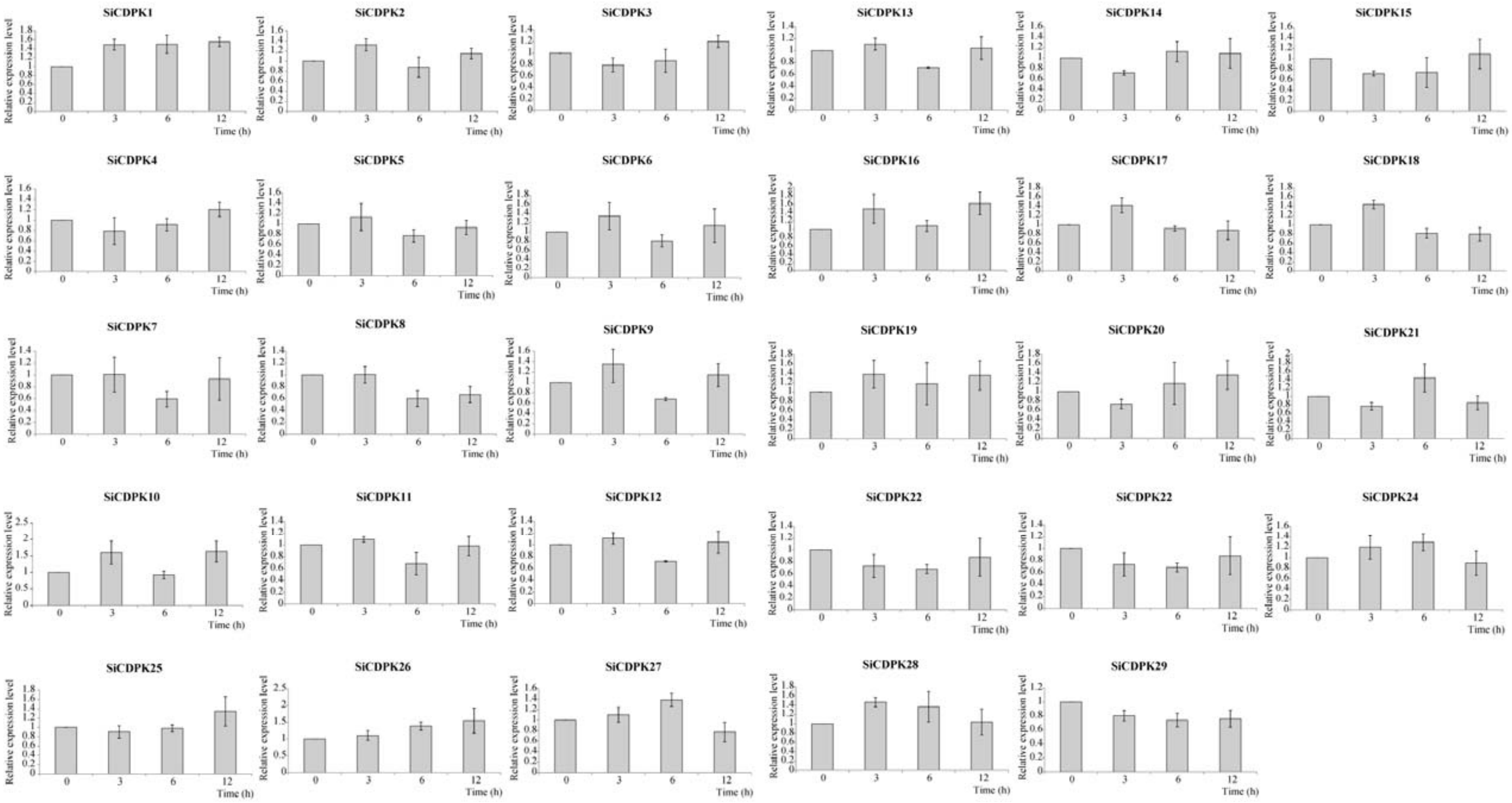

Figure S2 The expression levels of 29 foxtail millet CDPKs at different time period of H_2O treatment.
